# 基于二代测序技术分析骨髓纤维化患者基因突变对芦可替尼疗效的影响

**DOI:** 10.3760/cma.j.issn.0253-2727.2022.04.010

**Published:** 2022-04

**Authors:** 雅娴 谭, 洁 罗, 继贤 黄, 冬梅 罗, 瀚尹 梁, 璇 周, 晓力 刘, 娜 许

**Affiliations:** 1 南方医科大学南方医院血液科，广州 510515 Department of Hematology, Nan fang Hospital, Southern Medical University, Guangzhou 510515, China; 2 粤北人民医院血液科，韶关 512025 Department of Hematology, Yuebei People's Hospital, Shaoguan 512025, China

**Keywords:** 骨髓纤维化, 二代测序, 芦可替尼, 驱动基因, Myelofibrosis, Next-generation sequencing, Ruxolitinib, Driver mutation

## Abstract

**目的:**

评估基因突变对芦可替尼治疗骨髓纤维化（MF）疗效的影响。

**方法:**

回顾性分析2017年7月至2020年12月服用芦可替尼治疗并应用二代测序技术检测127个血液肿瘤相关基因突变的56例MF患者的临床资料，分析突变基因与芦可替尼疗效的关系。

**结果:**

①56例患者中，原发性骨髓纤维化（PMF）36例、真性红细胞增多症（PV）后骨髓纤维化（PPV-MF）9例，原发性血小板增多症（ET）后骨髓纤维化（PET-MF）11例。②50例（89.29％）携带驱动基因突变，22例（39.29％）携带基因突变≥3个，29例（51.79％）检出高危基因突变（HMR）。③对于基因突变≥3个的MF患者，芦可替尼仍有较好的改善体质性症状及缩小脾脏的效果（*P*＝0.001，*P*<0.001）。与基因突变<3个组比较，基因突变≥3个组停药前持续用药时间（TTF）及无进展生存期（PFS）明显缩短［356（55～1061）d对471.5（50～1270）d，*z*＝−2.701，*P*＝0.007；444（91～4109）d对1248.5（91～7061）d，*z*＝−2.030，*P*＝0.042］。④与未检出HMR的患者比较，≥2个HMR患者的芦可替尼缩脾效果较差（*t*＝10.471，*P*＝0.034），TTF及PFS明显缩短（*P*<0.001，*P*＝0.001）。⑤在携带ASXL1、EZH2、SRSF2等附加基因突变患者中，芦可替尼缩脾、症状改善及稳定骨髓纤维化作用较差，携带ASXL1、EZH2突变患者TTF［ASXL1：360（55～1270）d对440（55～1268）d，*z*＝−3.115，*P*＝0.002；EZH2：327（55～975）d对404（50～1270）d，*z*＝−3.219，*P*＝0.001］及PFS较未携带者明显缩短（ASXL1：457（50～1331）d对574（55～1437）d，*z*＝−3.219，*P*＝0.001；EZH2：428（55～1331）d对505（55～1437）d，*z*＝−2.576，*P*＝0.008］。

**结论:**

MF患者携带的基因突变类型、数量以及HMR对芦可替尼疗效有一定影响。

## Abstract (en)

**Objective:**

To assess the effect of gene mutations on the efficacy of ruxolitinib for treating myelofibrosis（MF）.

**Methods:**

We retrospectively analyzed the clinical data of 56 patients with MF treated with ruxolitinib from July 2017 to December 2020 and applied second-generation sequencing（NGS）technology to detect 127 hematologic tumor-related gene mutations. Additionally, we analyzed the relationship between mutated genes and the efficacy of ruxolitinib.

**Results:**

①Among the 56 patients, there were 36 cases of primary bone marrow fibrosis（PMF）, 9 cases of bone marrow fibrosis（ppv-mf）after polycythemia vera, and 11 cases of bone marrow fibrosis（PET-MF）after primary thrombocytosis（ET）. ②Fifty-six patients with MF taking ruxolitinib underwent NGS, among whom, 50（89.29％）carried driver mutations, 22（39.29％）carried ≥3 mutations, and 29（51.79％）carried high-risk mutations（HMR）. ③ For patients with MF carrying ≥ 3 mutations, ruxolitinib still had a better effect of improving somatic symptoms and shrinking the spleen（*P*＝0.001, *P*<0.001）, but TTF and PFS were significantly shorter in patients carrying ≥ 3 mutations（*P*＝0.007, *P*＝0.042）. ④For patients carrying ≥ 2 HMR mutations, ruxolitinib was less effective in shrinking the spleen than in those who did not carry HMR（*t*＝10.471, *P*＝0.034）, and the TTF and PFS were significantly shorter in patients carrying ≥2 HMR mutations（*P*<0.001, *P*＝0.001）. ⑤Ruxolitinib had poorer effects on spleen reduction, symptom improvement, and stabilization of myelofibrosis in patients carrying additional mutations in ASXL1, EZH2, and SRSF2. Moreover, patients carrying ASXL1 and EZH2 mutations had significantly shorter TTF［ASXL1: 360（55-1270）d *vs* 440（55–1268）d, *z*＝−3.115, *P*＝0.002; EZH2: 327（55–975）d *vs* 404（50–1270）d, *z*＝−3.219, *P*＝0.001］, and significantly shorter PFS compared to non-carriers［ASXL1: 457（50–1331）d *vs* 574（55–1437）d, *z*＝−3.219, *P*＝0.001）; 428（55–1331）d *vs* 505（55–1437）d, *z*＝−2.576, *P*＝0.008］.

**Conclusion:**

The type and number of mutations carried by patients with myelofibrosis and HMR impact the efficacy of ruxolitinib.

芦可替尼是首个批准用于治疗中、高风险原发性骨髓纤维化（PMF）、真性红细胞增多症（PV）后骨髓纤维化（PPV-MF）和原发性血小板增多症（ET）后骨髓纤维化（PET-MF）的JAK抑制剂。多项临床研究证实芦可替尼可使肿大的脾脏缩小、改善疾病相关体质性症状及提高生活质量，但其是否可以延缓克隆进展及逆转骨髓纤维化仍有争议[1]–[2]。在COMFORT-Ⅰ和COMFORT-Ⅱ研究中，随访3年后有不低于50％的患者因失去治疗反应、疾病进展或因治疗相关不良事件而停药[2]–[3]。既往，我们应用二代测序发现基因突变与MF的临床特征密切相关[2]–[3]。近年来研究发现，缺乏JAK2、MPL、CALR等驱动基因突变患者的白血病转化风险增加，总生存（OS）期缩短[5]。亦有研究发现MF患者存在ASXL1、EZH2、IDH1、IDH2和SRSF2等高危基因突变（HMR），并与预后不良相关[6]。但是国内相关研究相对较少。在本研究中，我们对2017年7月至2020年12月在南方医科大学南方医院血液科接受芦可替尼治疗并应用二代测序检测127个血液肿瘤相关基因突变的56例MF患者进行回顾性分析。

## 病例与方法

1. 病例资料：本研究纳入2017年7月至2020年12月在南方医院血液科规范服用芦可替尼的56例MF患者（PMF 36例，PPV-MF 9例，PET-MF 11例），所有患者均行二代测序，PMF的诊断符合《原发性骨髓纤维化诊断与治疗中国指南（2019年版）[7]，PPV-MF和PET-MF诊断则采用国际骨髓纤维化研究和治疗工作组（IWG-MRT）标准[8]。所有病例均进行动态国际预后评分系统（DIPSS）评分，均符合芦可替尼用药指证并且规律使用芦可替尼直至停药。所有患者在开始治疗前、使用芦可替尼12个月后分别行骨髓穿刺、血液肿瘤相关基因二代测序分析、脾脏超声测量及骨髓增殖性肿瘤症状评估表（MPN-10）[9]评分。

2. 血液肿瘤相关基因二代测序分析：采集56例患者骨髓样本，使用全自动核酸提取仪（厦门凯硕生物科技有限公司产品）提取gDNA并构建Illumina标准文库，NimbleGen液相杂交捕获芯片（瑞士罗氏公司产品）进行血液肿瘤相关的127个基因目标序列捕获，在Nextseq 550AR（美国Illumina公司产品）上进行PE75测序。分析内容包括确定点突变（SNV）、插入和缺失（INDEL）、内部串联重复（ITD）和部分串联重复（PTD）等变异类型。使用MuTect2软件检测SNV和INDEL变异，ITD和PTD的变异检测通过Illumina公司自主研发算法完成。变异检测结果采用Annovar软件进行注释。为保证变异准确率，对原始变异检测结果进行过滤：每个样本捕获目标区域平均有效深度≥1 000×，支持突变型的reads比对质量和碱基质量值均高于30，并且同时具有正负链支持。

3. 随访及观察终点：①停药前持续用药时间（TTF）：定义为从开始芦可替尼治疗到治疗中断或患者死亡的时间。②无进展生存期（PFS）：定义为从开始使用芦可替尼到疾病发生进展之间的时间。③脾脏反应：采用超声技术测量患者脾脏长径来评估脾脏大小，观察芦可替尼治疗后患者脾脏长径变化。④临床症状改善：观察芦可替尼治疗前后患者体质性症状（疲劳、虚弱、腹痛、恶病质、体重减轻、瘙痒、盗汗和骨痛），使用MPN-10症状评估表评估MF患者症状严重程度。采用查阅门诊/住院病历和电话随访方式获得患者病情资料。

4. 统计学处理：采用SPSS 23.0软件进行数据分析。符合正态分布计量资料的比较采用单因素方差分析或*t*检验，非正态分布计量资料的比较使用Kruskal-Wallis *H*检验或Mann-Whitney *U*检验；计数资料采用卡方检验进行差异性分析，检验水平α＝0.05；若三组间差异有统计学意义，再分别进行两两比较，采用Bonferroni法进行校正，校正后检验水平α＝0.017；使用Spearman Correlation检验两组计数资料的相关性。

## 结果

1. 患者一般资料：纳入本研究的56例患者包括PMF 36例，PET-MF 11例，PPV-MF 9例，所有纳入的病例均为DIPSS中危-2、高危组患者。三组患者的基线指标差异均无统计学意义（表1），所有患者的中位随访时间为782（91～7061）d。截止到2020年12月31日，有39例患者仍在服用芦可替尼，所有患者芦可替尼治疗的中位时间为422（50～1270）d。PMF、PET-MF、PPV-MF患者的TTF分别为380（50～1270）d、559（119～1248）d、574（55～1001）d（*H*＝3.714，*P*＝0.156），PFS分别为684（91～5235）d、1674（274～6727）d、1036（91～7061）d（*H*＝1.397，*P*＝0.497）。

**表1:** 56例骨髓纤维化患者芦可替尼治疗前临床资料及芦可替尼起始剂量

临床特征	PMF（36例）	PET-MF（11例）	PPV-MF（9例）	统计量	*P*值
性别（例，男/女）	19/17	6/5	4/5	*χ*^2^＝0.242	0.886
年龄［岁，*M*（范围）］	66.5（36～79）	63（44～84）	60（24～68）	*H*＝0.317	0.854
WBC［×10^9^/L，*M*（范围）］	19.84（1.23～222.01）	22.67（7.01～80.18）	18.82（7.87～33.00）	*H*＝2.571	0.276
HGB［g/L，*M*（范围）］	94（59～137）	115（70～174）	197（109～238）	*H*＝0.286	0.867
PLT［×10^9^/L，*M*（范围）］	167（5～607）	369（161～1084）	464（117～630）	*H*＝3.429	0.180
外周血原始细胞比例［％，*M*（范围）］	1（0～10）	1（0～3）	0（0～2）	*H*＝0.250	0.882
脾脏长径［cm，*M*（范围）］	19.4（10.0～27.2）	16.1（9.8～21.2）	14.7（12.2～23.1）	*H*＝4.571	0.102
脾脏厚径［cm，*M*（范围）］	6.5（4.3～10.5）	5.9（2.9～8.7）	5.0（4.2～7.2）	*H*＝4.321	0.109
MPN10评分［分，*M*（范围）］	20（18～59）	18（4～43）	17（0～57）	*H*＝0.000	1.000
骨髓纤维化（网染）［*M*（范围）］	1+（0～3+）	1+（0～3+）	3+（1+～ 4+）	*H*＝0.250	0.882
异常核型（例）	4	0	0	*χ*^2^＝2.393	0.302
输血依赖（例）	4	1	1	*χ*^2^＝0.038	0.981
芦可替尼起始剂量（例）					
≤20 mg/d	11	3	4	*χ*^2^＝0.786	0.675
> 20 ～<40 mg/d	10	4	2	*χ*^2^＝0.516	0.773
≥40 mg/d	15	4	3	*χ*^2^＝0.259	0.879

注：PMF：原发性骨髓纤维化；PPV-MF：真性红细胞增多症后骨髓纤维化；PET-MF：原发性血小板增多症后骨髓纤维化；三阴性：JAK2、CALR和MPL基因突变均为阴性；MPN-10：骨髓增殖性肿瘤症状评估表；TTF：停药前持续用药时间

2. 二代测序结果：56例患者中，37例（66.07％）检出JAK2基因突变、9例（16.07％）检出CALR基因突变、5例（8.93％）检出MPL基因突变，6例（10.71％）患者JAK2、CALR、MPL基因突变均未检出（三阴性），其中2例（3.57％）未检测到基因突变。所有患者共检出129例次基因突变，平均每例患者携带2.30个基因突变，其中29例携带HMR（PMF患者23例，PPV-MF患者5例，PET-MF患者1例）。检测到突变频率大于5的附加基因依次为ASXL1 19例（33.93％）、TET2 13例（23.21％）、EZH2 8例（14.29％）、SRSF2 5例（8.93％）。

3. 携带总突变基因数量对芦可替尼疗效的影响：根据患者携带的突变基因数量，我们将56例患者分为突变基因<3个（34例）及突变基因≥3个（22例）两组，两组初诊时各项指标基线临床数据差异均无统计学意义。经芦可替尼治疗后，两组患者的脾脏大小及MPN10评分均有明显好转（表2），其中突变基因≥3个组芦可替尼治疗后MPN10评分较初诊时明显下降［11.5（4～18）分对21.5（4～57）分，*z*＝−3.411，*P*＝0.001］，治疗后脾脏长径较治疗前明显缩短［15.2（10.2～25.8）cm对16.0（10.4～27.0）cm，*z*＝−4.117，*P*<0.001］，表明芦可替尼对于突变基因≥3个的MF患者仍有改善体质性症状及缩小脾脏的效果。对比两组患者治疗后的脾脏大小发现，携带的基因突变数对芦可替尼缩脾作用无明显影响（*H*＝0.000，*P*＝0.993）（表2）。突变基因≥3个组中有4例患者治疗后骨髓纤维化程度较治疗前加重，而基因突变<3个组中有2例患者骨髓纤维化程度较治疗前加重，两组间差异无统计学意义（*H*＝0.001，*P*＝0.972）。突变基因≥3个组TTF及PFS明显缩短［356（55～1061）d对471.5（50～1270）d，*z*＝−2.701，*P*＝0.007；444（91～4109）d对1248.5（91～7061）d，*z*＝−2.030，*P*＝0.042］。PFS曲线见图1。

**表2:** 基因突变<3个与≥3个两组骨髓纤维化（MF）患者的临床数据比较

临床特征	突变数<3个（34例）	突变数≥3个（22例）	统计量	*P*值
性别（例，男/女）	15/19	8/14	*χ*^2^＝0.332	0.565
年龄［岁，*M*（范围）］	58.0（24～84）	62.5（42～79）	*H*＝3.888	0.040
WBC［×10^9^/L，*M（*范围）］	15.75（1.23～80.18）	21.96（5.67～222.01）	*H*＝3.631	0.046
HGB［g/L，*M*（范围）］	105（34～215）	96（58～238）	*H*＝1.208	0.272
PLT［×10^9^/L，*M*（范围）］	277.5（30～1451）	206（29～900）	*H*＝1.153	0.283
外周血原始细胞比例［％，*M*（范围）］	1.0（0～10）	1.5（0～6.5）	*H*＝0.345	0.557
异常核型（例）	1	3	*χ*^2^＝0.996	0.318
脾脏长径［cm，*M*（范围）］				
治疗前	17.6（9.8～26.4）	16.0（10.4～27.0）	*H*＝0.077	0.782
治疗后	13.4（9.5～22.5）^b^	15.2（10.2～25.8）^b^	*H*＝0.001	0.993
统计量	*z*＝−4.723	*z*＝−4.117		
*P*值	<0.001	<0.001		
MPN10［分，*M*（范围）］				
治疗前	18（0～59）	21.5（4～57）	*H*＝0.793	0.373
治疗后	13（0～38）^b^	11.5（4～18）^b^	*H*＝0.902	0.342
统计量	z＝−2.853	*z*＝−3.411		
*P*值	0.004	0.001		
骨髓纤维化分级［*M*（范围）］				
治疗前	3+（1+～4+）	3+（1+～ 4+）	*H*＝1.364	0.243
治疗后	2+（0～4+）^b^	2+（0～3+）^a^	*H*＝0.001	0.972
统计量	*z*＝−4.056	*z*＝−2.506		
*P*值	<0.001	0.012		
芦可替尼起始剂量（例）				
≤20 mg/d	10	8	*χ*^2^＝0.296	0.586
> 20～<40 mg/d	9	7	*χ*^2^＝0.187	0.665
≥40 mg/d	15	7	*χ*^2^＝0.847	0.357
芦可替尼减量/停药（例）	7	9	*χ*^2^＝2.703	0.100

注：MPN10：骨髓增殖性肿瘤症状评估表。^a^ 与治疗前比较，*P*<0.05；^b^ 与治疗前比较，*P*<0.01

**图1:**
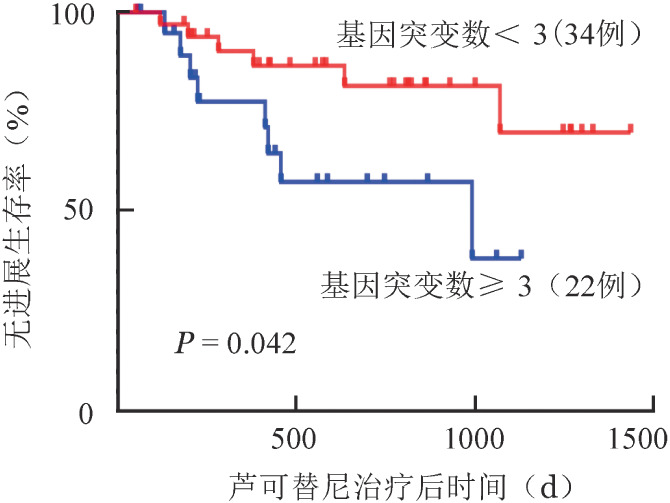
基因突变数量对骨髓纤维化患者芦可替尼治疗后无进展生存的影响

4. HMR对芦可替尼疗效的影响：56例MF患者中，17例检出1个HMR，9例检出≥2个HMR，30例未检出HMR，三组患者芦可替尼治疗前的各项临床数据差异均无统计学意义（*P*>0.05，表3）。未检出HMR、1个HMR、≥2个HMR三组患者经芦可替尼治疗后脾脏均明显缩小（*z*＝−4.412，*P*<0.001；*z*＝−3.420，*P*＝0.001；*z*＝−2.668，*P*＝0.008），治疗后三组患者的脾脏长度差异具有统计学意义（*H*＝6.101，*P*＝0.047），两两比较显示芦可替尼对于≥2个HMR组的缩脾效果劣于无HMR突变组（*t*＝10.471，*P*＝0.034）。芦可替尼治疗后，未检出HMR、1个HMR、≥2个HMR三组的MPN10评分均较治疗前明显下降（*z*＝−2.894，*P*＝0.004；*z*＝−2.407，*P*＝0.016；*z*＝−2.310，*P*＝0.021），三组间的MPN10评分比较在治疗前后均无明显差异。三组患者的骨髓纤维化程度在治疗前后无明显变化（*z*＝−0.175，*P*＝0.861；*z*＝−0.061，*P*＝0.952；*z*＝−0.378，*P*＝0.705），治疗期间三组患者骨髓纤维化程度加重的例数分别为2、3、1例，治疗后三组间骨髓纤维化程度差异无统计学意义（*H*＝0.717，*P*＝0.397），详见表4。无HMR、1个HMR、≥2个HMR组TTF分别为665（119～1270）d、283（50～1061）d、121（55～587）d（*H*＝13.897，*P*<0.001），PFS分别为754（119～1437）d、413（50～1300）d、203（55～587）d（*H*＝14.433，*P*＝0.001）（图2）。在22例突变基因≥3个MF患者中，有9例（40.91％）携带≥2个HMR突变，使用Spearman Correlation检验两组变量的相关性，显示两组数据间无明显相关性（*r*＝−0.013，*P*＝0.927）。

**表3:** 携带不同高危基因突变（HMR）的骨髓纤维化患者基础临床数据

临床特征	无HMR（30例）	1个HMR（17例）	≥2个HMR（9例）	统计量	*P*值
性别（例，男/女）	13/17	8/9	8/1	*χ^2^*＝3.632	0.163
年龄［岁，*M*（范围）］	58.5（24～84）	61（38～79）	62（42～78）	*H*＝1.297	0.523
WBC［×10^9^/L，*M*（范围）］	18.02（1.23～80.18）	15.70（2.90～63.27）	23.70（5.67～222.01）	*H*＝1.621	0.445
HGB［g/L，*M*（范围）］	110（63～215）	87（34～201）	93（59～238）	*H*＝4.634	0.099
PLT［×10^9^/L，*M*（范围）］	318（30～1451）	214（56～900）	119（29～639）	*H*＝3.884	0.143
外周血原始细胞比例［％，*M*（范围）］	0.75（0～10）	0.5（0～3.5）	1.0（0～3.5）	*H*＝0.551	0.759
异常核型（例）	1	1	2	*χ^2^*＝3.783	0.151
芦可替尼起始剂量					
≤20 mg/d	11	3	4	*χ^2^*＝2.544	0.280
>20～<40 mg/d	6	8	2	*χ^2^*＝4.105	0.128
40 mg/d	13	6	3	*χ^2^*＝0.453	0.797
芦可替尼减量/停药（例）	7	6	4	*χ^2^*＝1.714	0.419

**表4:** 高危基因突变（HMR）对骨髓纤维化患者芦可替尼疗效的影响

临床特征	无HMR（30例）	1个HMR（17例）	≥2个HMR（9例）	统计量	*P*值
脾脏长径［cm，*M*（范围）］					
治疗前	16.8（9.8～26.4）	17.0（10.4～27.0）	19.4（10.0～26.3）	*H*＝2.267	0.123
治疗后	13.0（9.5～22.5）	13.5（10.2～19.0）	16.5（10.0～22.0）	*H*＝6.101	0.047
统计量	*z*＝−4.412	*z*＝−2.668	*z*＝−3.420		
*P*值	<0.001	0.008	0.001		
MPN10［分，*M*（范围）］					
治疗前	18（2～59）	21（0～57）	25（4～55）	*H*＝1.639	0.441
治疗后	11.5（0～38）	13（4～18）	11（8～15）	*H*＝0.176	0.916
统计量	*z*＝−2.894	*z*＝−2.407	*z*＝−2.310		
*P*值	0.004	0.016	0.021		
骨髓纤维化［*M*（范围）］					
治疗前	3+（1+～3+）	3+（0～3+）	3+（1+～ 3+）	*H*＝0.506	0.477
治疗后	3+（0～3+）	2+（1+～3+）	2+（2+～3+）	*H*＝0.717	0.397
统计量	*z*＝−0.175	*z*＝−0.061	*z*＝−0.378		
*P*值	0.861	0.952	0.705		

注：MPN10：骨髓增殖性肿瘤症状评估表

**图2:**
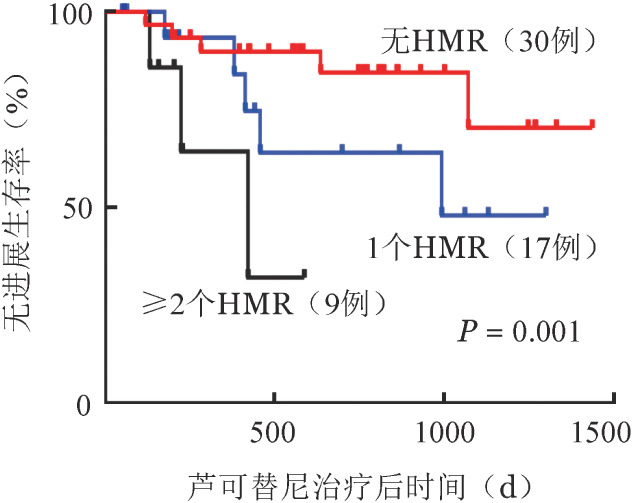
高危基因突变（HMR）数量对骨髓纤维化患者芦可替尼治疗后无进展生存的影响

5. 附加基因突变对芦可替尼疗效的影响：我们比较了携带/未携带高频率突变的附加基因（大于5例患者检出该基因突变）ASXL1、TET2、EZH2、SRSF2的患者芦可替尼治疗前后的临床资料，结果显示芦可替尼对于携带SRSF2突变的MF患者缩脾效果较差［治疗前13.6（10.0～21.4）cm，治疗后12.8（10.0～19.8）cm，*z*＝−1.826，*P*＝0.068］，携带EZH2、SRSF2突变的MF患者症状改善效果较差［EZH2组治疗前、后MPN10积分分别为20.5（4～35）分、11.5（8～16）分，*z*＝−1.825，*P*＝0.068；SRSF2组治疗前、后MPN10积分分别为16（9～55）分、11（8～13）分，*z*＝−1.761，*P*＝0.078］，携带ASXL1、EZH2、SRSF2突变的MF患者稳定骨髓纤维化作用较差（表5）。携带、不携带ASXL1突变患者TTF分别为360（55～1270）d、440（55～1268）d（*z*＝−3.115，*P*＝0.002），PFS分别为457（50～1331）d、574（55～1437）d（*z*＝−3.219，*P*＝0.001）；携带、不携带EZH2突变患者TTF分别为327（55～975）d、404（50～1270）（*z*＝−3.219，*P*＝0.001），PFS分别为428（55～1331）d、505（55～1437）（*z*＝−2.576，*P*＝0.008）；TET2、SRSF2突变对MF患者芦可替尼TTF、PFS无显著影响（表6）。

**表5:** 附加基因突变对骨髓纤维化患者芦可替尼疗效的影响［*M*（范围）］

附加基因	例数	脾脏长径（cm）				MPN10评分（分）				骨髓纤维化分级			
		治疗前	治疗后	*z*值	*P*值	治疗前	治疗后	*z*值	*P*值	治疗前	治疗后	*z*值	*P*值
ASXL1													
有	19	19.4（10.4~27.0）	17.5（10.2~25.8）	−3.831	<0.001	18（2~59）	11.5（0~38）	−2.418	0.016	3+（1+~3+）	2+（0~3+）	−1.933	0.053
无	37	16.6（9.8~26.4）	13.3（9.5~22.5）	−5.078	<0.001	18（2~59）	12（0~38）	−3.427	0.001	3+（0~3+）	2+（1+~3+）	−4.018	<0.001
TET2													
有	13	16.5（9.8~23.1）	14.8（9.5~22.5）	−3.062	0.002	15（2~55）	9（0~18）	−2.075	0.038	3+（0+~ 3+）	1+（0~3+）	−2.157	0.031
无	43	17.6（9.8~27.0）	14.3（9.5~25.8）	−5.578	<0.001	20（2~59）	13（0~38）	−3.538	<0.001	3+（0~3+）	2+（0~3+）	−3.928	<0.001
EZH2													
有	8	20.2（13.5~27.0）	19.4（12.8~25.8）	−2.524	0.012	20.5（4~35）	11.5（8~16）	−1.825	0.068	3+（1+~3+）	3+（0~3+）	−0.577	0.564
无	48	16.8（9.8~24.6）	13.8（9.5~20.6）	−5.814	<0.001	18（2~59）	12.5（0~38）	−3.654	<0.001	3+（0~3+）	2+（0~3+）	−4.395	<0.001
SRSF2													
有	5	13.6（10.0~21.4）	12.8（10.0~19.8）	−1.826	0.068	16（9~55）	11（8~13）	−1.761	0.078	3+（2+~3+）	2+（1~2+）	−1.857	0.063
无	51	17.6（9.8~27.0）	14.6（9.5~25.8）	−6.083	<0.001	19（2~59）	13（0~38）	−3.782	<0.001	3+（0~3+）	2+（0~3+）	−4.051	<0.001

注：MPN10：骨髓增殖性肿瘤症状评估量表

**表6:** 附加基因突变对骨髓纤维化患者芦可替尼停药前持续用药时间（TTF）和无进展生存期（PFS）的影响［d，*M*（范围）］

附加基因	例数	TTF			PFS		
		值	*z*值	*P*值	值	*z*值	*P*值
ASXL1			−3.115	0.002		−3.219	0.001
有	19	360（55～1270）			457（50～1331）		
无	37	440（55～1268）			574（55～1437）		
TET2			−0.243	0.808		−0.107	0.915
有	13	413（55～1061）			440（55～1331）		
无	43	383（50～1270）			553（50～1437）		
EZH2			−3.219	0.001		−2.576	0.008
有	8	327（55～975）			428（55～1331）		
无	48	404（50～1270）			505（55～1437）		
SRSF2			−0.675	0.500		−0.503	0.615
有	5	269（71～930）			413（185～1437）		
无	51	395（50～1270）			553（50～1437）		

7. 长期随访及生存：有11例患者在随访期中死亡（PMF 9例，PET-MF 1例，PPV-MF 1例），死亡原因包括急变（7例）、肠穿孔（1例）、消化道出血（1例）、心血管事件（1例）、感染（1例）。所有患者的芦可替尼中位治疗剂量为30（5～40）mg/d。17例患者在随访期间经历了芦可替尼减量及停药，减药/停药的原因为血液学不良反应（11例）、骨髓造血干细胞移植（4例）、经济原因（3例）、感染（3例）、腹水（1例）。

## 讨论

芦可替尼通过抑制MPN患者过度激活的JAK/STAT信号通路，缩小患者肿大的脾脏和改善症状，已经作为中、高危MF患者的一线用药，但JAK2的突变状态并不影响芦可替尼对于MPN患者的症状改善作用[10]。COMFORT-Ⅱ研究[11]发现，芦可替尼对具有不同基因突变的MPN患者脾脏和症状反应相似，并且JAK抑制剂不会降低急性白血病转化的风险。三阴性患者占所有MPN患者的10％～15％，OS期仅为2.4年并且有着更高的白血病转化风险[12]–[13]。与携带驱动基因突变患者相比，三阴性患者的白细胞计数、血红蛋白浓度水平和血栓发生率较低，并且年龄较低[14]。随着对MPN致病机制研究的深入，目前研究发现，除JAK2、CALR、MPL等驱动基因突变之外的附加突变在MPN的发生起重要作用，其中ASXL1、EZH2、SRSF2、IDH1、IDH2这5种基因被定义为与白血病转化相关的HMR，而发生附加突变的数目直接与预后和并发症的发生相关，频繁的基因突变也与PMF患者持续的体质性症状相关[15]–[16]。本研究发现芦可替尼对携带附加基因突变患者仍有缩脾及改善体质性症状疗效，但携带ASXL1及EZH2突变患者的TTF及PFS均明显缩短，其他附加基因突变因检出频次较少未行统计学分析。与COMFORT-Ⅱ中所得的结果相似[17]，在我们的研究中，对于携带≥2个HMR突变的患者，芦尼可替缩脾的效果较差。而对于突变数量对芦可替尼疗效的影响，与先前的研究不一致的是[11]，本研究中携带突变数≥3个的MF患者芦可替尼仍具有较好的缩脾效果。经过长期随访，我们发现携带HMR突变及突变数≥3个的患者TTF及PFS明显缩短。

国外一些研究显示芦可替尼3年间的停药率约为50％[11]，停用芦可替尼后患者的中位OS期仅为14个月[18]。芦可替尼停药的主要原因是脾脏反应缺乏或减弱（22.9％、11.9％）以及疾病进展（23.4％）。在服用芦可替尼过程中应关注疾病克隆演化、药物不良反应及治疗期间的感染防控。

本研究结果显示，MF患者携带的基因突变类型、数量以及HMR对芦可替尼疗效有一定影响。二代基因测序检测MF患者驱动基因和附加基因突变可能有助于预测芦可替尼疗效，为二线治疗方案的制订提供依据。
